# Calibration of Yld2000-2D Anisotropy Yield Criterion with Traditional Testing and Inverse Identification Strategies

**DOI:** 10.3390/ma16216904

**Published:** 2023-10-27

**Authors:** Jiaqi Chen, Zhihao Wang, Xingrong Chu, Zhenming Yue, Chao Zhao, Yiqi Zhou

**Affiliations:** 1 Key Laboratory of High-Efficiency and Clean Mechanical Manufacture, Ministry of Education, Shandong University, Jinan 250061, China; jiaqi_chen@mail.sdu.edu.cn (J.C.); yqzhou@sdu.edu.cn (Y.Z.); 2 School of Mechanical Engineering, Shandong University, Jinan 250061, China; 3 School of Mechanical, Electrical and Information Engineering, Shandong University, Weihai 264209, China; zhihaow_edu@163.com (Z.W.); yuezhenming@sdu.edu.cn (Z.Y.); tabianshijie@126.com (C.Z.)

**Keywords:** plastic anisotropy, yield criterion, biaxial tensile test, parameter calibration, inverse identification

## Abstract

In order to improve predictive capabilities of numerical simulations, Yld2000-2D yield criterion is used to model the plastic anisotropic behaviors of AA5086 sheets. The parameters of Yld2000-2D yield criterion are identified based on the traditional testing strategy and the inverse identification strategy, respectively. The traditional testing strategy considers uniaxial and equi-biaxial tensile tests. The inverse identification strategy relies on the finite element model update (FEMU) method that couples with a biaxial tensile test using a dedicated cruciform specimen or the Pottier bulging test. The identified parameters are preliminarily evaluated by comparing predicted and experimental yield stresses, *r*-values, and yield loci. Then, the deep drawing test and simulations are performed. The identified parameter sets of Yld2000-2D yield criterion are further evaluated in terms of practical forming by comparing the predicted earing profile height with the experimental results. The results show that the inverse identification strategy can be an effective alternative to identify the parameters of Yld2000-2D yield criterion, and a well-designed heterogeneous test could lead to a better identification result.

## 1. Introduction

Aluminum alloy is widely used in aerospace, shipbuilding, and automobile manufacturing due to its excellent properties such as low density and high corrosion resistance [1,2]. However, aluminum alloy usually exhibits plastic anisotropy after thermomechanical treatments. Therefore, modeling its anisotropy is essential for accurately predicting the mechanical behavior of its sheets in advanced manufacturing engineering. Over the past few decades, plenty of anisotropy yield criteria have been proposed, such as Hill’48 [3], Yld2000-2D [4,5], Yld2004 [6], and Bron–Besson yield criterion [7]. Hill’48 is a classical one and is widely used in finite element (FE) simulations of sheet metals due to the simplicity of its parameter identification. While for some advanced anisotropy yield criteria with many parameters, parameter identification is an ongoing need. Currently, the parameter identification procedure can be classified into two main strategies: traditional testing and inverse identification.

The traditional testing strategy is based on some homogeneous deformation tests, such as uniaxial tensile tests, shear tests, etc., to accurately analyze the mechanical properties of sheet metals under well-defined stress states. For the identification of anisotropic yield criterion parameters, the required mechanical properties mainly include the material orientation-dependent yield stresses and *r*-values. For example, the parameter identification of the Yld2000-2D yield criterion can be performed using uniaxial tensile tests at 0°, 45°, and 90° to the rolling direction, as well as an equi-biaxial tensile test [4]. Zang et al. [8] characterized the yield stresses and *r*-values of mild and dual-phase steel sheets by performing shear, uniaxial, and biaxial tensile tests. These results were then used to identify the parameters of the Bron–Besson yield criterion. With a similar identification strategy, Zhang et al. [9] performed the parameter identification of the Bron–Besson yield criterion at three plastic strain levels to consider the evolution of material anisotropy with plastic strain. Performing equi-biaxial tensile test requires a dedicated biaxial tensile equipment, which is usually expensive and technically complex. An alternative is to substitute it with other tests. Tian et al. [10] employed a disk compression test to determine the *r*-value under the equi-biaxial tension for calibrating the Yld2000-2D yield criterion. Commonly, for the traditional testing strategy, the number of experimental campaigns should not be less than the number of parameters to be identified. However, when the experimental data are insufficient to determine the parameters of the yield criterion, it would be acceptable to compensate the data by reasonable assumptions (e.g., using isotropic parameters) or by numerical predictions based on advanced microstructural models. Khalfallah et al. [11] identified the parameters of Cazacu and Barlat yield criterion (CB2001) using a reduced set of experimental data combined with some artificially generated data.

The inverse identification strategy is a full-field measurement-based approach that extracts more information from non-standard tests to retrieve anisotropic yield criterion parameters. The full-field measurement usually relies on the Digital Image Correlation (DIC) technique. The non-standard test usually employs a well-designed specimen to generate a heterogeneous deformation field. Due to the heterogeneity, various regions of the specimen experience different stress states and strain paths, and thus, a wealth of information about material anisotropy can be extracted from a single test [12]. From this perspective, the inverse identification strategy shows a high potential to simplify the experimental campaigns without sacrificing the identification accuracy [13]. The inverse identification strategy combined with non-standard tests for material parameter identification has been rapidly developed in recent years and is called Materials Testing 2.0 [14], promising to revamp traditional testing. Avril et al. [15] presented a complete overview and comparison of several mainstream methods of the inverse identification strategy. Among them, the FEMU [16] is a more intuitive approach. The principle of the FEMU is to minimize the gap between numerical predictions and experimental measurements by iteratively updating the model parameters. Numerical predictions are obtained from the reproduction of the experiment using an FE model. The experimental measurements can be either full-field measurements or partial measurements of the full-field. The mapping relationship between the prediction and experiment can be established based on displacement, strain, force, etc. The FEMU is a mature technique with high robustness and low sensitivity to measurement noise, and it is capable of modeling complex specimen geometries [17]. Based on the FEMU method, Pottier et al. [18] identified the parameters of Hill’48 yield criterion using a well-designed specimen. The specimen was out of plane deformed to generate significant heterogeneous deformation fields with tensile, shear, and expansion strain states. The identified parameters were verified using the deep drawing test, and the results showed that the inverse identification combined with heterogeneous strain field can better predict the actual deformation compared to traditional tests. Wang et al. [19] used the same specimen for testing at an elevated temperature and identified the parameters of Yld2000-2D criterion for 7B04 aluminum alloy at 200 °C. The biaxial tensile test is a special case of multi-axial loading. By using a well-designed cruciform specimen, a heterogeneous test can also be achieved. Zhang et al. [20] designed a cruciform specimen with notches to supply experimental data for the inverse procedure. Based on this, the parameters of Bron and Besson yield criterion were accurately identified. Martins et al. [21] used a biaxial test and the Virtual Fields Method (VFM) to calibrate the parameters of Yld2000-2D criterion and Swift’s hardening law. 

The present paper focuses on the parameter identification of Yld2000-2D anisotropy yield criterion of AA5086 sheets using traditional testing and inverse identification strategies. The aim is to provide a detailed insight into the implementation and validation of these two strategies and to provide a comprehensive comparison. The traditional testing method considers three uniaxial tensile tests at different orientations and an equi-biaxial tensile test. The inverse identification strategy relies on the FEMU method coupling with a biaxial tensile test using a dedicated cruciform specimen or the Pottier bulging test. To verify the identified anisotropy parameters, a comparison between the experimental and Yld2000-2D-predicted yield stresses, *r*-values, and yield loci is performed. In addition, a deep drawing test is carried out. The identified yield criterion is further evaluated in terms of practical forming by comparing the predicted earing height distribution with the experimental results. All specimens in this work are extracted from 2 mm thick AA5086 sheets using laser cutting.

## 2. Yld2000-2D Anisotropy Yield Criterion

Yld2000-2D yield criterion proposed by Barlat et al. [4] can accurately describe the anisotropic behavior of aluminum alloy sheets. The function is as follows:

(1) $$\Psi ={\left[\frac{1}{2}\left(\Psi^{\prime }+\Psi^{\prime \prime }\right)\right]}^{\frac{1}{m}}$$

(2) $$\Psi^{\prime }={\left|X_{1}^{{}^{\prime }}+X_{2}^{{}^{\prime }}\right|}^{m}$$

(3) $$\Psi^{\prime \prime }={\left|2X_{2}^{{}^{\prime \prime }}+X_{1}^{{}^{\prime \prime }}\right|}^{m}+{\left|2X_{1}^{{}^{\prime \prime }}+X_{1}^{{}^{\prime \prime }}\right|}^{m}$$

(4) $$X_{i}^{{}^{\prime }}=\frac{1}{2}\left(X_{11}^{{}^{\prime }}+X_{22}^{{}^{\prime }}\pm \sqrt{{\left(X_{11}^{{}^{\prime }}-X_{22}^{{}^{\prime }}\right)}^{2}+4X_{12}^{{}^{\prime }}{}^{2}}\right)$$

(5) $$X_{j}^{{}^{\prime \prime }}=\frac{1}{2}\left(X_{11}^{{}^{\prime \prime }}+X_{22}^{{}^{\prime \prime }}\pm \sqrt{{\left(X_{11}^{{}^{\prime \prime }}-X_{22}^{{}^{\prime \prime }}\right)}^{2}+4X_{12}^{{}^{\prime \prime }}{}^{2}}\right)$$

(6) $$X^{\prime }=L^{\prime }\sigma \;X^{\prime \prime }=L^{\prime \prime }\sigma$$

where *m* is a material parameter, set as eight in this study, which depends on the crystal structure of AA5086. $X_{i}^{{}^{\prime }}$ and $X_{j}^{{}^{\prime \prime }}$ are the principal values of the tensors $X^{\prime }$ and $X^{\prime \prime }$. ***σ*** is Cauchy stress. $L^{\prime }$ and $L^{\prime \prime }$ are expressed as follows ($\alpha_{1}\sim \alpha_{8}$ are eight anisotropy parameters to be identified):

(7) $$\left[\begin{array}{c} L_{11}^{{}^{\prime }}\\ L_{12}^{{}^{\prime }}\\ L_{21}^{{}^{\prime }}\\ L_{22}^{{}^{\prime }}\\ L_{66}^{{}^{\prime }} \end{array}\right]=\left[\begin{array}{ccc} 2/3 & 0 & 0\\ -1/3 & 0 & 0\\ 0 & -1/3 & 0\\ 0 & 2/3 & 0\\ 0 & 0 & 1 \end{array}\right]\left[\begin{array}{c} \alpha_{1}\\ \alpha_{2}\\ \alpha_{7} \end{array}\right]$$

(8) $$\left[\begin{array}{c} L_{11}^{{}^{\prime \prime }}\\ L_{12}^{{}^{\prime \prime }}\\ L_{21}^{{}^{\prime \prime }}\\ L_{22}^{{}^{\prime \prime }}\\ L_{66}^{{}^{\prime \prime }} \end{array}\right]=\frac{1}{9}\left[\begin{array}{ccccc} -2 & 2 & 8 & -2 & 0\\ 1 & -4 & -4 & 4 & 0\\ 4 & -4 & -4 & 1 & 0\\ -2 & 8 & 2 & -2 & 0\\ 0 & 0 & 0 & 0 & 9 \end{array}\right]\left[\begin{array}{c} \alpha_{3}\\ \alpha_{4}\\ \alpha_{5}\\ \alpha_{6}\\ \alpha_{8} \end{array}\right]$$

## 3. Parameter Identification with Traditional Testing Strategy

For the traditional testing strategy, yield stresses and *r*-values obtained from uniaxial and equi-biaxial tensile tests are used to identify the parameters of Yld2000-2D yield criterion. For all the tests in this study, strain fields are measured with the DIC system. Two high-resolution CCD cameras (1624 × 1236 pixels) are used to capture the images, and the acquisition frequency is set to 20 images/s. The subset size of 16 pixels and the step size of 8 pixels are adopted for the strain calculation.

### 3.1. Uniaxial and Biaxial Tensile Tests

Aluminum–magnesium alloys (5xxx series) are widely used in automotive, aircraft, and naval industries due to their high strength-to-weight ratio, high corrosion resistance, and good workability characteristics. The used material’s (AA5086) chemical components are shown in Table 1.

**Figure 1:**
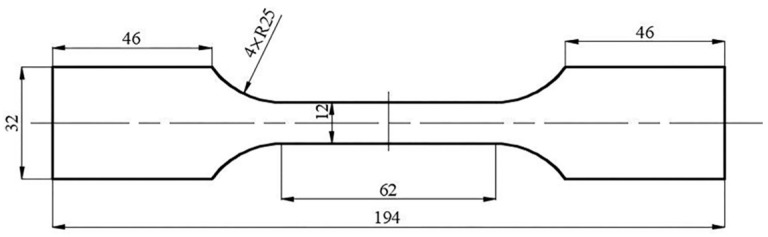
Dimensions of uniaxial tensile specimen (unit: mm).

**Figure 2:**
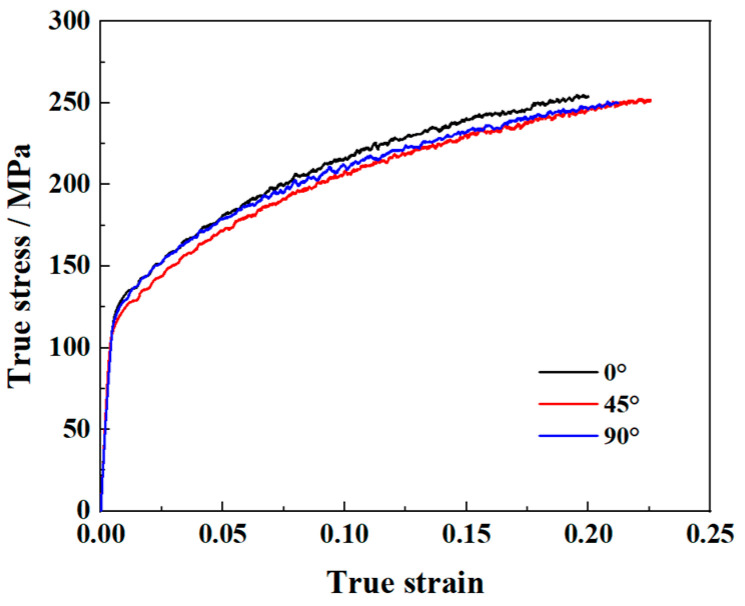
True stress–strain curves of AA5086 sheets from uniaxial tensile tests.

The dimensions of the uniaxial tensile specimen are shown in Figure 1. Uniaxial tensile tests are performed at 0°, 45°, and 90° to the rolling direction (RD). The tensile velocity is set to 0.372 mm/min, which corresponds to a strain rate of 10^−4^ s^−1^. Each test is performed three times to ensure repeatability of the results. Figure 2 only presents one curve per direction for a better view. The determined yield stresses and *r*-values (*r* = *ε_w_*/*ε_t_*, the ratio of plastic strain in width and thickness directions) of AA5086 sheets are summarized in Table 2. The material hardening behavior is described using a Voce law [22]:

(9) $$\bar{\sigma }=\sigma_{0}+k\left(1-\exp\left(-n\cdot {\bar{\varepsilon }}^{p}\right)\right)$$

where *σ*_0_ is the initial yield stress of material in RD. ${\bar{\varepsilon }}^{p}$ donates the equivalent plastic strain. *k* and *n* are material parameters. By fitting the Voce law to the true stress-equivalent plastic strain curve (obtained from the uniaxial tensile test along RD), the *k* and *n* are calibrated to be 150.4 and 10.7, respectively.

**Figure 3:**
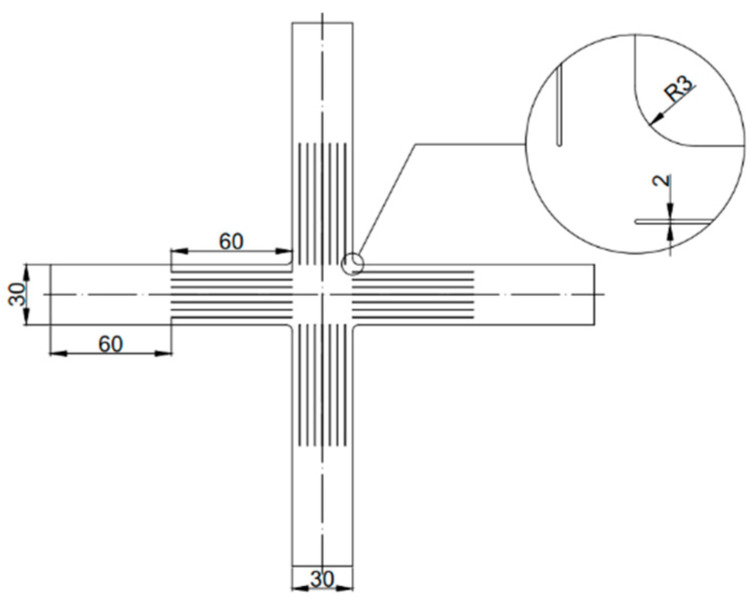
The dimensions of cruciform specimen used for equi-biaxial yield stress and *r*-value determination (unit: mm).

The cruciform specimen (in Figure 3), according to the ISO 16842:2021 [23], is employed to determine the yield stress *σ_b_* and the anisotropy coefficient *r_b_* with an equi-biaxial tensile test. The specimen central rectangular zone (21 × 21 mm^2^) is selected as the strain calculation area. Three tests are performed to ensure the repeatability of results. The yield stress *σ_b_* is calculated to be 106.155 MPa, 106.259 MPa, and 105.787 MPa, respectively. The average value (106.067 MPa) is adopted for the parameter identification. In addition, to determine the anisotropy coefficient *r_b_*, the employed formula [4] is as follows:

(10) $$r_{b}=\frac{\varepsilon_{yy}}{\varepsilon_{xx}}$$

where *ε_xx_* and *ε_yy_* are the strain components along RD and transverse direction (TD), respectively. By performing linear fitting of the slopes of *ε_xx_* versus *ε_yy_*, the *r_b_* from three equi-biaxial tensile tests are calculated as 1.123, 1.109, and 1.072, respectively. Then, an average value (1.101) is adopted for the parameter identification.

### 3.2. Parameter Identification of Yld2000-2D Yield Criterion

The principle of the traditional testing strategy for the Yld2000-2D parameters identification is minimizing the gap between experimental yield stresses and *r*-values and theoretical calculations. The gap is quantified with a cost function, which is given by Equation (11). The identification procedure is performed using the software mode-FRONTIER and MATLAB. The MOSA (Multi Objective Simulated Annealing) optimization algorithm is employed to find the optimal set of the parameters to be identified.

(11) $$\delta ={\sum_{i=1}^{3}\left(\frac{\sigma_{i}^{cal}-\sigma_{i}^{\exp}}{\sigma_{i}^{\exp}}\right)}^{2}+{\left(\frac{\sigma_{b}^{cal}-\sigma_{b}^{\exp}}{\sigma_{b}^{\exp}}\right)}^{2}+0.45\times \left[\sum_{i=1}^{3}{\left(\frac{r_{i}^{cal}-r_{i}^{\exp}}{r_{i}^{\exp}}\right)}^{2}+{\left(\frac{r_{b}^{cal}-r_{b}^{\exp}}{r_{b}^{\exp}}\right)}^{2}\right]$$

where *σ^cal^* and *σ^exp^* denote the yield stresses of theoretical calculation and experiment, respectively. *r^cal^* and *r^exp^* denote the *r*-values of theoretical calculation and experiment, respectively. *i* is an index indicating the material orientation. A weight coefficient of 0.45 is added to the *r*-value term with the aim of improving the fitting of yield stress. 

**Figure 4:**
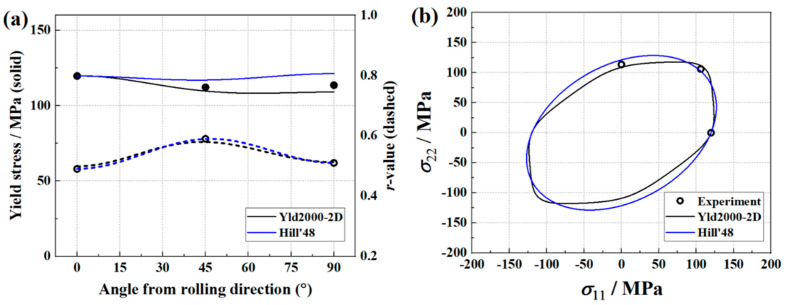
Comparison of Hill’48 and Yld2000-2D yield criterion. (**a**) Yield stresses, *r*-values, and (**b**) yield loci.

The identified Yld2000-2D parameters are provided in Table 3, and Figure 4 shows the experimental and Yld2000-2D-predicted yield stresses and *r*-values, as well as the plane stress yield locus. In addition, the results predicted by Hill’48 anisotropy yield criterion are also presented in this figure. The Hill’48 parameters (in Table 4) are calculated based on the *r*-values. It is found that the Yld2000-2D yield criterion provides accurate predictions for yield stresses and *r*-values, while Hill’48 fails to predict the yield stresses at 45 and 90 degrees. For the yield locus, the Yld2000-2D yield criterion also provides a better prediction. Therefore, using an advanced yield criterion contributes to the accurate prediction of anisotropy behaviors of AA5086 sheets.

## 4. Parameter Identification with Inverse Identification Strategy

**Figure 5:**
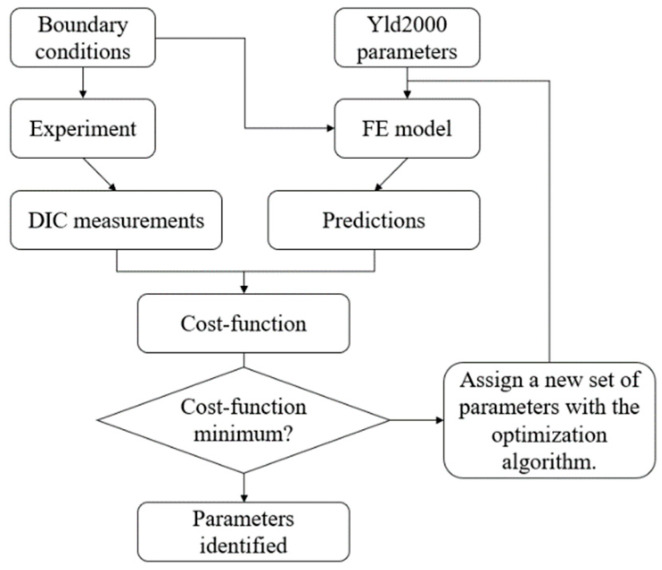
Flowchart of the FEMU method for the inverse identification of Yld2000-2d parameters.

In this section, the inverse identification strategy based on the FEMU method is implemented to determine the Yld2000-2D parameters of AA5086 sheets. An equi-biaxial tensile test using the dedicated cruciform specimen [9] and the Pottier bulging test [18] are performed to produce heterogeneous deformation fields and to provide experimental data for parameter identification. The process flowchart of the FEMU method is shown in Figure 5.

### 4.1. Identification with Equi-Biaxial Tensile Test

**Figure 6:**
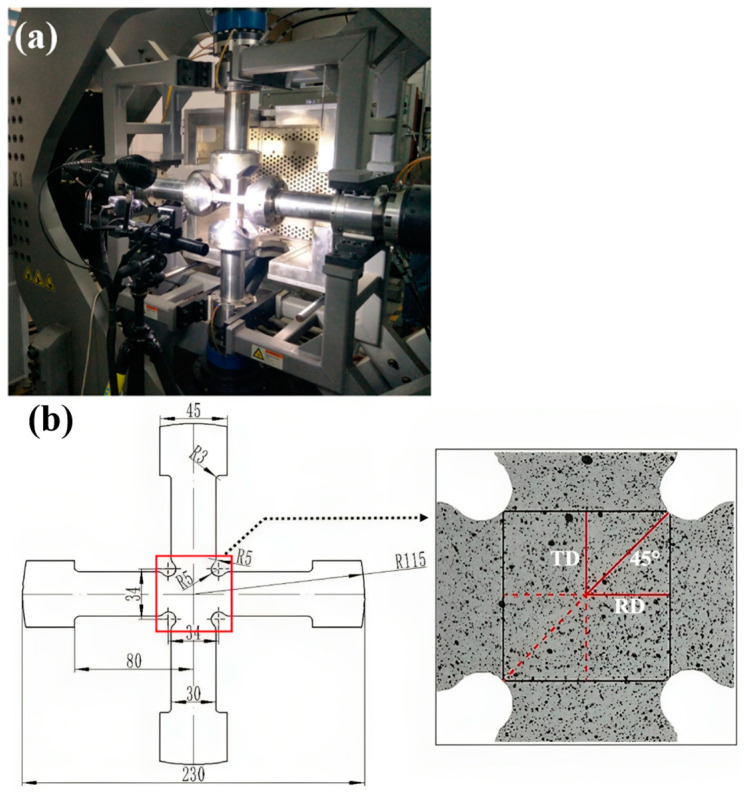
(**a**) The device for the biaxial tensile test with 3D-DIC system, and (**b**) dimensions of the cruciform specimen and the ROI.

**Figure 7:**
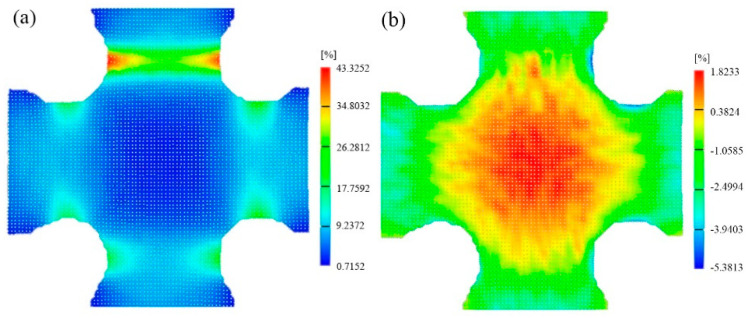
Principal strain fields of the specimen’s ROI: (**a**) major strain field and (**b**) minor strain field.

The device for the biaxial tensile test and the dimensions of the cruciform specimen are shown in Figure 6. During the test, each arm of the specimen is stretched at a constant velocity of 0.1 mm/s, and the force of the two tensile axes are recorded. The specimen’s central area (30 mm × 30 mm) is selected as the region of interest (ROI). The local kinematic measurements of the major and minor strain fields in the ROI are implemented through the DIC system. The major and minor strain fields in the ROI just before the occurrence of fracture (time point: 79.1 s) are provided in Figure 7. It is observed that the strain state in the ROI is significantly heterogeneous. From the specimen’s center to its boundary area, the strain state transitions from equi-biaxial tension to uniaxial tension via a plane strain state. A localized necking area can be found near the specimen notches, with the specimen center reaching a lower strain level, which is a typical feature of this kind of test [21]. Considering that the mechanical properties of the material after necking are significantly affected due to the damage accumulation, and the constitutive model used in this work does not involve material damage behaviors, the strain fields at the time point of 75 s before the onset of localized necking are adopted for the inverse identification procedure.

**Figure 8:**
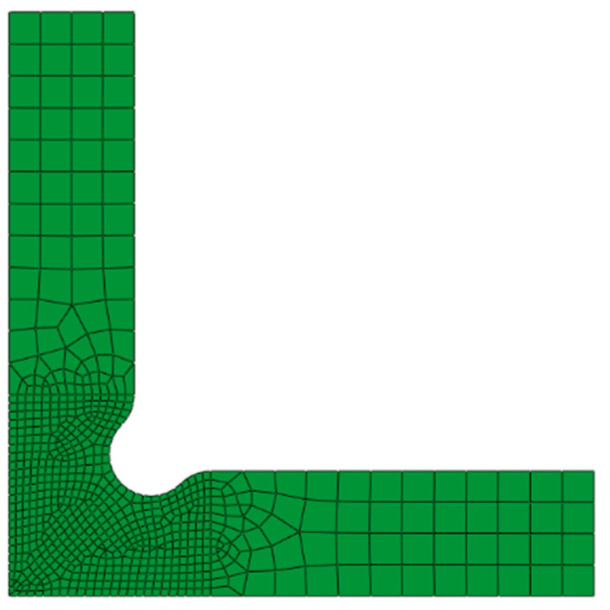
FE model of the cruciform specimen.

The principle of the FEMU method is to minimize the gap between the numerical predictions and experimental measurements by iteratively updating the parameters to be identified. The predictions are obtained with the FE model of the biaxial tensile test, which is built using software ABAQUS v6.14/Standard. As shown in Figure 8, only a quarter of the cruciform specimen is modeled considering the symmetry. Experimental force data from the beginning to the time point of 75 s are imposed on the corresponding arms of the specimen. The specimen is discretized through four node shell elements with a minimum size of 1 mm. The mesh size is determined with a convergence check, with particular attention provided to its effect on the major and minor strains. The Yld2000-2D anisotropy yield criterion with Voce law is implemented through a user subroutine UMAT. 

According to the research conducted by Zhang et al. [9], the major and minor strains extracted from the points along the 45° direction of ROI are used as experimental data for the inverse identification. In addition, the major and minor strains extracted from the elements along the 45° direction in the predicted results of the simulated step of 75 s are used as prediction data. Due to the coordinate deviation of the points used for strain extraction in the FE model and the experiment, a linear interpolation is performed on the experimental strain data. Then, the gap between numerical and experimental major and minor strains is quantified with a cost function:

(12) $$\delta ={\sum_{i=1}^{n}\left(\frac{\varepsilon_{\mathrm{major},\;i}^{\mathrm{sim}}-\varepsilon_{\mathrm{major},\;i}^{\exp}}{\varepsilon_{\mathrm{major},\;i}^{\exp}}\right)}^{2}+{\sum_{i=1}^{n}\left(\frac{\varepsilon_{\mathrm{minor},\;i}^{\mathrm{sim}}-\varepsilon_{\mathrm{minor},\;i}^{\exp}}{\varepsilon_{\mathrm{minor},\;i}^{\exp}}\right)}^{2}$$

where *i* is an index indicating the sample number, and the total number of samples is 20.

**Figure 9:**
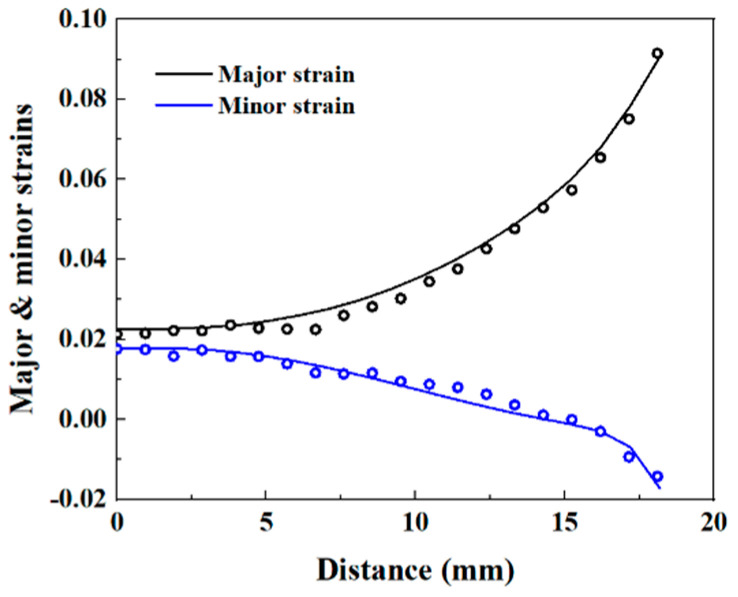
Yld2000-2D-predicted (lines) and experimental (symbols) major and minor strains of material points along the 45° direction of the specimen’s ROI.

**Figure 10:**
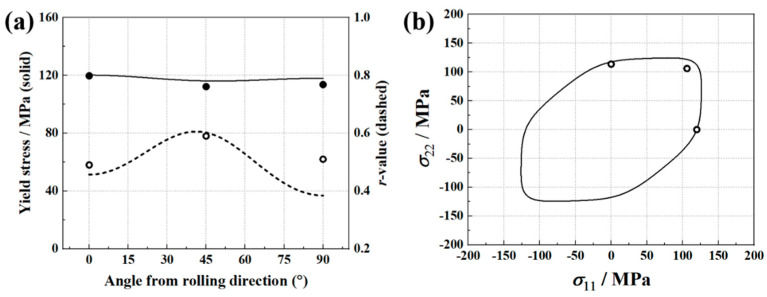
(**a**) Experimental (symbols) and Yld2000-2D-predicted yield stresses (solid lines) and *r*-values (dashes lines). (**b**) Yld2000-2D-predicted yield locus. The Yld2000-2D parameters according to Table 5.

The implementation of FEMU is carried out through the mode-FRONTIER platform, and the update of the parameters is achieved by coupling the SIMPLEX optimization algorithm with the cost function. The Uniform Latin Hypercube algorithm is adopted to define nine initial sets of parameters, which allows them to be equally spaced over the parameter ranges. The identified parameters of the Yld2000-2D anisotropy yield criterion are shown in Table 5. Based on the determined parameters, the predicted major and minor strains are in good agreement with the experimental results, as shown in Figure 9. In addition, Figure 10 presents a comparison between predicted yield stresses, *r*-values, and yield locus and experimental results. The identified Yld2000-2D yield criterion provides a correct prediction of the material yield stresses, while the predicted *σ_b_* is slightly higher than the experimental one. For the *r*-values, the predictions are close to the experimental values in the 0° and 45° directions, but significantly lower in the 90° direction. The deviation in the predicted 90° *r*-values could be attributed to the experimental measurements used in the FEMU procedure that did not sufficiently cover the anisotropic behavior of the material, as only the principal strains extracted from the 45° path of the specimen’s ROI were considered. The use of multiple paths of the ROI in the FEMU procedure to fully cover the material anisotropy is recommended. Martins et al. [21] used similar tests and the VFM method to inverse identify the Yld2000-2D parameters for an aluminum alloy and a mild steel. The identified Yld2000-2D also showed a deviation in the prediction of r-values for the aluminum alloy, while r-values of the mild steel were correctly predicted. A conclusion was drawn that this approach is sensitive to the material used.

### 4.2. Identification with Pottier Bulging Test

**Figure 11:**
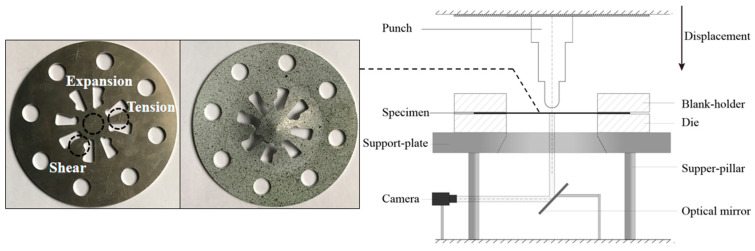
Pottier specimens before and after the bulging test, and schematic diagram of the test device.

**Figure 12:**
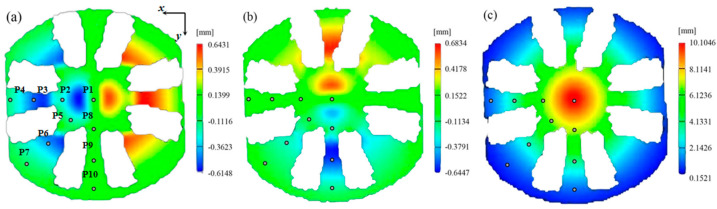
Experimentally measured displacement fields at time point of 100 s: (**a**) *U_x_*, (**b**) *U_y_*, and (**c**) *U_z_*. Positions of the 10 points for extracting the displacement data.

The specimen and the schematic diagram of the Pottier bulging test device are shown in Figure 11. The diameter of the specimen is 130 mm, and 15 mm for the hemispherical punch. Two cameras are employed in the DIC system to measure the out-of-plane displacements of the specimen. The images of the specimen’s bottom surface are captured through an optical plane mirror placed below the specimen. The displacement velocity of the punch is 0.1 mm/s. The specimen can exhibit the tensile, shear, and expansion behaviors of the material. The displacement fields of the specimen at the time point of 100 s (just before the localized necking occurs) are used as experimental data, and the FEMU method is then adopted to identify the parameters of Yld2000-2D yield criterion. Figure 12 presents the measured displacement fields (*u_x_*, *u_y_*, and *u_z_*) and the positions of 10 points for extracting the displacement data. 

For the FE model of the Pottier bulging test, the ROI of the specimen is meshed by three node shell elements with a size of 1 mm. The specimen thickness is defined based on the specimen’s bottom surface, and five thickness integration points are adopted. The simulation is performed by applying the same velocity and stroke to the punch as in the test. In the simulation analysis step of 100 s, the displacements are extracted from the elements corresponding to the positions of the ten experimental points and used as prediction data. A linear interpolation of the experimental data is performed according to the positions of the elements. The gap between numerical and experimental displacements is quantified with a cost function:

(13) $$\delta ={\sum_{i=1}^{n}\left(\frac{U_{x,i}^{\mathrm{sim}}-U_{x,i}^{\exp}}{U_{x,i}^{\exp}}\right)}^{2}+{\sum_{i=1}^{n}\left(\frac{U_{y,i}^{\mathrm{sim}}-U_{y,i}^{\exp}}{U_{y,i}^{\exp}}\right)}^{2}+{\sum_{i=1}^{n}\left(\frac{U_{z,i}^{\mathrm{sim}}-U_{z,i}^{\exp}}{U_{z,i}^{\exp}}\right)}^{2}$$

where *i* is an index indicating the sample number, and the total number of samples is 10. *U_x_*, *U_y_*, and *U_z_* are the displacements along *x*, *y*, and *z* directions, respectively. The rolling direction of the material corresponds to the *x* direction.

**Figure 13:**
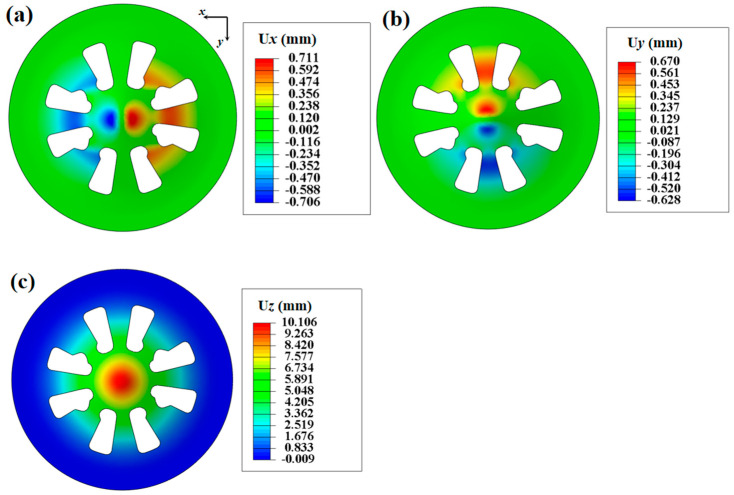
Predicted displacement fields based on the identified Yld2000-2D yield criterion: (**a**) *U_x_*, (**b**) *U_y_*, and (**c**) *U_z_*.

**Figure 14:**
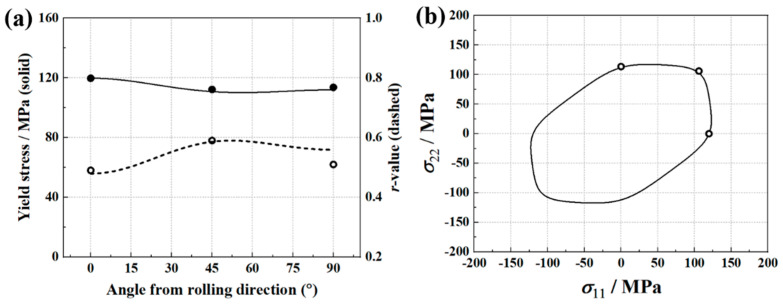
(**a**) Experimental (symbols) and Yld2000-2D-predicted yield stresses (solid lines) and *r*-values (dashes lines). (**b**) Yld2000-2D-predicted yield locus. The Yld2000-2D parameters according to Table 6.

The FEMU method with SIMPLEX optimization algorithm is then used to minimize the gap, and the identified parameters of the Yld2000-2D yield criterion are provided in Table 6. The predicted displacement fields based on these parameters are shown in Figure 13. It is observed that the identification procedure leads to a good match between the predicted and experimental displacement fields. In addition, Figure 14 presents a comparison between predicted yield stresses, *r*-values, and yield locus and experimental results. The Yld2000-2D yield criterion accurately captures the yield stresses of AA5086 sheets in different material orientations and in the equi-biaxial tension state. The predicted *r*-value is slightly overestimated for the 90° direction compared to the experimental one. 

## 5. Verification of Yield Criterion Parameters

**Figure 15:**
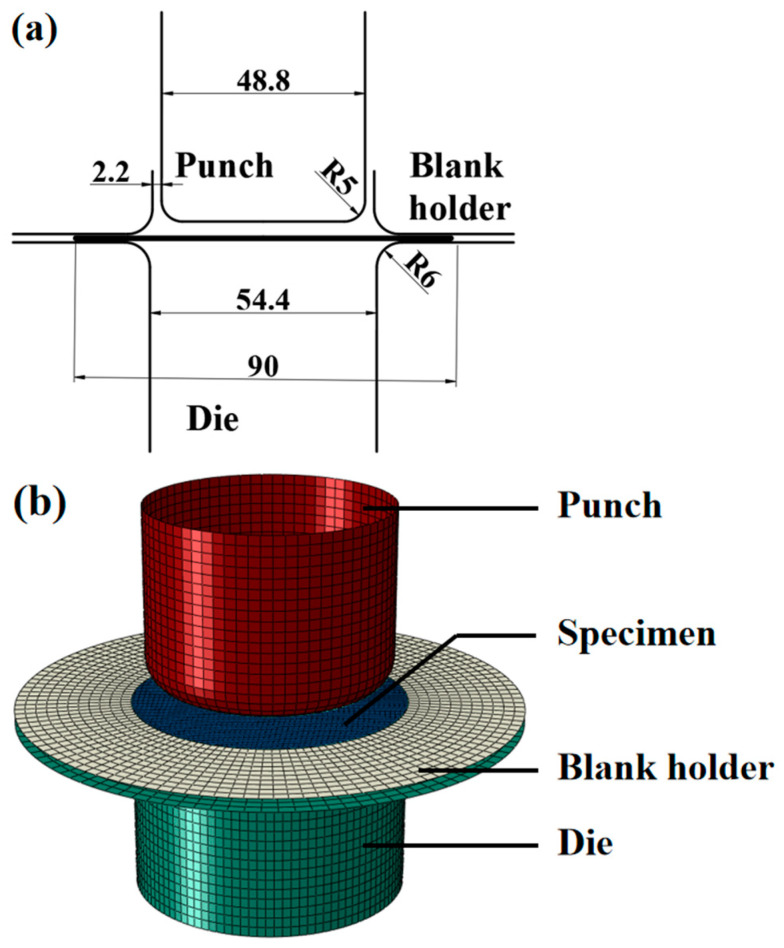
(**a**) Schematic diagram of the deep drawing test device (unit: mm) and (**b**) the FE model.

Plastic anisotropy is the main factor leading to earing behavior in the deep drawing test. In order to verify the accuracy of the Yld2000-2D anisotropy yield criterion parameters identified by different methods, a deep drawing test is carried out. The shape of the specimen is circular, with a diameter of 90 mm and a thickness of 2 mm. The schematic diagram of the deep drawing device is shown in Figure 15a. The punch speed is set as 0.01 mm/s, and a 6 kN blank holder force provided by two nitrogen springs is adopted. Lubrication is applied to the specimen, the punch, and the die. The earing height distribution of formed specimen is measured using a height gauge. The FE model of the deep drawing test is shown in Figure 15b. The specimen is modeled with four node shell elements, and the punch, the die, and the blank holder are modeled using the discrete rigid bodies. The friction coefficient between the specimen and the punch and the die is set to 0.2. The numerical deep drawing test is then performed with the identified parameter sets of Yld2000-2D yield criterion and stopped at the corresponding experimental drawing depth (65 mm).

**Figure 16:**
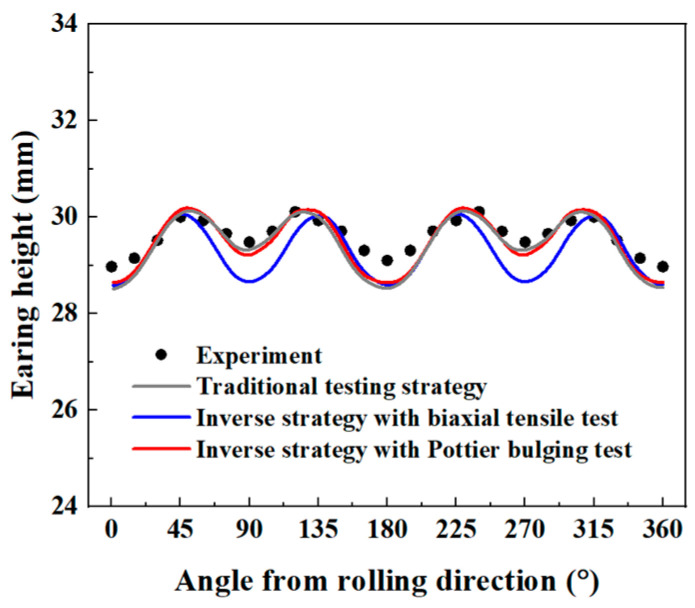
Predicted earing height distributions in comparison with the experiment.

The experimental and predicted earing profiles are presented in Figure 16. The anisotropy of AA5086 sheets leads to four ears, and the peaks of the earing profile appear at about 45°, 135°, 225°, and 315° directions. This distribution is similar to the results presented by Neto et al. [24]. Considering the test symmetry, here only the earing profiles at 0 ° and 180° are discussed. It is found that the identified Yld2000-2D parameter sets from both the traditional testing and inverse identification strategies accurately predicted the heights and locations of the earing profile peaks. For the valleys of 0° and 180° directions, the three Yld2000-2D parameter sets provide similar predictions of the earing height, but all are about 1.7% below the experimental value. In addition, a significant difference between the three predicted earing profiles is observed around the 90° direction. With Figure 4, Figure 10, and Figure 14, one may notice that there is also a significant difference in the *r*_90_ predicted by the three Yld2000-2D parameter sets. The parameter sets identified using the traditional testing and inverse identification (Pottier bulging test) strategies successfully captured *r*_90_ of AA5086 sheets, and therefore, the prediction of earing height in the 90° direction is accurate. While the *r*_90_ predicted with Yld2000-2D parameters from the inverse identification strategy (biaxial tensile test) is significantly lower than the experimental one, so it provides an underestimated prediction of earing height in the 90° direction. Compared to the heterogeneous deformation field from the biaxial tensile test, the one from the Pottier bulging test provided more information on the inverse identification procedure, especially on the material deformation under shear loading. Therefore, it is revealed that the quality and richness of the information provided by the heterogeneous tests could lead to better identification of Yld2000-2D parameters.

In summary, the parameters of Yld2000-2D yield criterion identified based on the traditional testing strategy can accurately describe the anisotropy behaviors of AA5086 sheets as expected. The inverse identification strategy based on the FEMU method and heterogeneous tests is also an effective alternative to identify the Yld2000-2D parameters with satisfactory accuracy. In addition, the capacity to predict the practical forming process is improved when test heterogeneity increases. The inverse identification strategy eliminates the need for a large number of experiments as required by the traditional testing strategy, and a single heterogeneous test allows the simultaneous identification of eight parameters of Yld2000-2D yield criterion.

## 6. Conclusions

In this study, the traditional testing and inverse identification strategies are used to identify the parameters of Yld2000-2D anisotropy yield criterion for AA5086 sheets. The traditional testing strategy adopts four yield stresses and *r*-values from uniaxial and equi-biaxial tensile tests. The inverse identification strategy is based on the FEMU method. The heterogeneous strain fields from the biaxial tensile test and the heterogeneous displacement fields from the Pottier bulging test are used to supply experimental data for the inverse identification, respectively. The identified parameter sets of Yld2000-2D anisotropy yield criterion are evaluated using the yield stresses, *r*-values, and yield loci, as well as the earing profile height of a deep drawing test. The main conclusions are as follows:

1. The traditional testing strategy can accurately identify the parameters of Yld2000-2D anisotropy yield criterion. The identified Yld2000-2D accurately describes the yield stresses and *r*-values of AA5086 sheets. The prediction of the earing height is also in good agreement with the experimental result.
2. The equi-biaxial tensile test using a dedicated cruciform specimen for producing heterogeneous strain fields has been investigated. The inverse identification of Yld2000-2D parameters is performed by minimizing the gap between the experimental and numerical principal strains along a diagonal direction of the specimen’s ROI. The identified Yld2000-2D parameters provide a correct prediction of the material yield stresses. But for the *r*-values at the 90° direction, the predicted values are significantly lower than the experiment. This results in predicted earing heights for the deep drawing test being lower than the experimental measurements in the 90° and 270° directions.
3. The Pottier bulging test for producing heterogeneous displacement fields has also been investigated. The inverse identification procedure of Yld2000-2D parameters is performed based on displacement data extracted from 10 material points covering the expansion, shear, and tension regions of the Pottier specimen. The identified Yld2000-2D parameters accurately describe the material anisotropic behaviors (yield stresses and *r*-values), and the predicted earing height distribution is close to the experimental one.
4. The inverse identification strategy based on the FEMU method can be an effective alternative to identify the parameters of Yld2000-2D yield criterion. A well-designed heterogeneous test could lead to a better identification result.

## Figures and Tables

**Table 1:** Chemical components of AA5086 in weight precent (wt.%).

Si	Fe	Cu	Mn	Mg	Cr	Zn	Ti	Al
0.32	0.41	0.06	0.51	4.12	0.19	0.16	0.07	Other

**Table 2:** Experimental data of AA5086 sheets from uniaxial and biaxial tensile tests.

*σ*_0_ (MPa)	*σ*_45_ (MPa)	*σ*_90_ (MPa)	*σ_b_* (MPa)	*r*_0_	*r*_45_	*r*_90_	*r_b_*
119.704	112.215	113.646	106.067	0.49	0.59	0.51	1.101

**Table 3:** Parameters of Yld2000-2D yield criterion identified with traditional testing strategy.

α_1_	α_2_	α_3_	α_4_	α_5_	α_6_	α_7_	α_8_
0.862	1.136	1.029	1.110	1.069	1.180	1.042	1.160

**Table 4:** Parameters of Hill’48 yield criterion identified with *r*-values.

F	G	H	N
0.645	0.671	0.329	1.434

**Table 5:** Yld2000-2D parameters identified with biaxial tensile test.

α_1_	α_2_	α_3_	α_4_	α_5_	α_6_	α_7_	α_8_
0.945	0.920	0.986	1.047	1.030	1.008	0.986	1.104

**Table 6:** Yld2000-2D parameters identified using Pottier bulging test.

α_1_	α_2_	α_3_	α_4_	α_5_	α_6_	α_7_	α_8_
0.8684	1.139	1.216	1.119	1.086	1.252	1.047	1.067

## Data Availability

Not applicable.
